# Pediatric Congenital External Auditory Canal Cholesteatoma Extending Beyond the External Auditory Canal: A Case Report

**DOI:** 10.7759/cureus.95546

**Published:** 2025-10-27

**Authors:** Kohei Yamahara, Takayuki Okano, Kana Sano, Ichiro Tateya

**Affiliations:** 1 Department of Otolaryngology-Head and Neck Surgery, School of Medicine, Fujita Health University, Toyoake, JPN; 2 Department of Otolaryngology-Head and Neck Surgery, School of Medicine, Fujita Health University Bantane Hospital, Nagoya, JPN

**Keywords:** acquired eacc, computed tomography, congenial eacc, differentiation between congenital and acquired eacc, pediatric external auditory canal cholesteatoma

## Abstract

This article describes an extremely rare case of pediatric congenital external auditory canal cholesteatoma (EACC) that extended beyond the external auditory canal. A five-year-old girl presented with progressive swelling in the posterior wall of the left external auditory canal. Computed tomography of the temporal bone revealed a well-defined round mass that compressed and eroded the posterior canal wall without invasion of the mastoid tegmen, sigmoid sinus, or tympanic membrane. Surgical exploration via a retroauricular approach confirmed the presence of a cholesteatoma extending from the external auditory canal to the mastoid cavity; furthermore, complete excision was achieved. There has been a recent increase in the number of reported EACC pediatric cases, especially in East Asia. However, few studies have clearly distinguished congenital and acquired forms, which could be largely attributed to challenges in differential diagnosis, particularly when lesions extend beyond the canal. Based on our findings, we propose radiological and clinical features that may facilitate differentiation between congenital and acquired EACC, even in advanced-stage cases. This article highlights the importance of accurate classification for elucidation of the pathogenesis of EACC and optimization of surgical decision-making in pediatric patients with EACC.

## Introduction

Cholesteatoma is characterized by the accumulation of desquamated keratin debris within the stratified squamous epithelium; furthermore, it may erode bone structures [1]. External auditory canal cholesteatoma (EACC) predominantly affects the elderly; however, there has been a gradual increase in reports of EACCs in pediatric patients. Generally, cholesteatomas are classified as acquired or congenital. Congenital cholesteatomas can arise in various regions of the temporal bone, including the external auditory canal [2-4], with the middle ear cavity being the most common site of origin. However, congenital EACC is extremely rare, with only two pediatric cases having been reported in the literature to date [4,5]. In pediatric cases, acquired EACC typically results from repeated microtrauma, while congenital EACC originates from the epithelial rests of embryonic tissue within the external auditory canal [6,7]. Extension of a congenital EACC beyond the external auditory canal with rupture of the skin surface impedes differentiation between congenital and acquired EACC. This article describes a pediatric case of suspected congenital EACC that extended beyond the external auditory canal. Furthermore, we propose an approach for differentiating between congenital and acquired EACC.

## Case presentation

**Figure 1:**
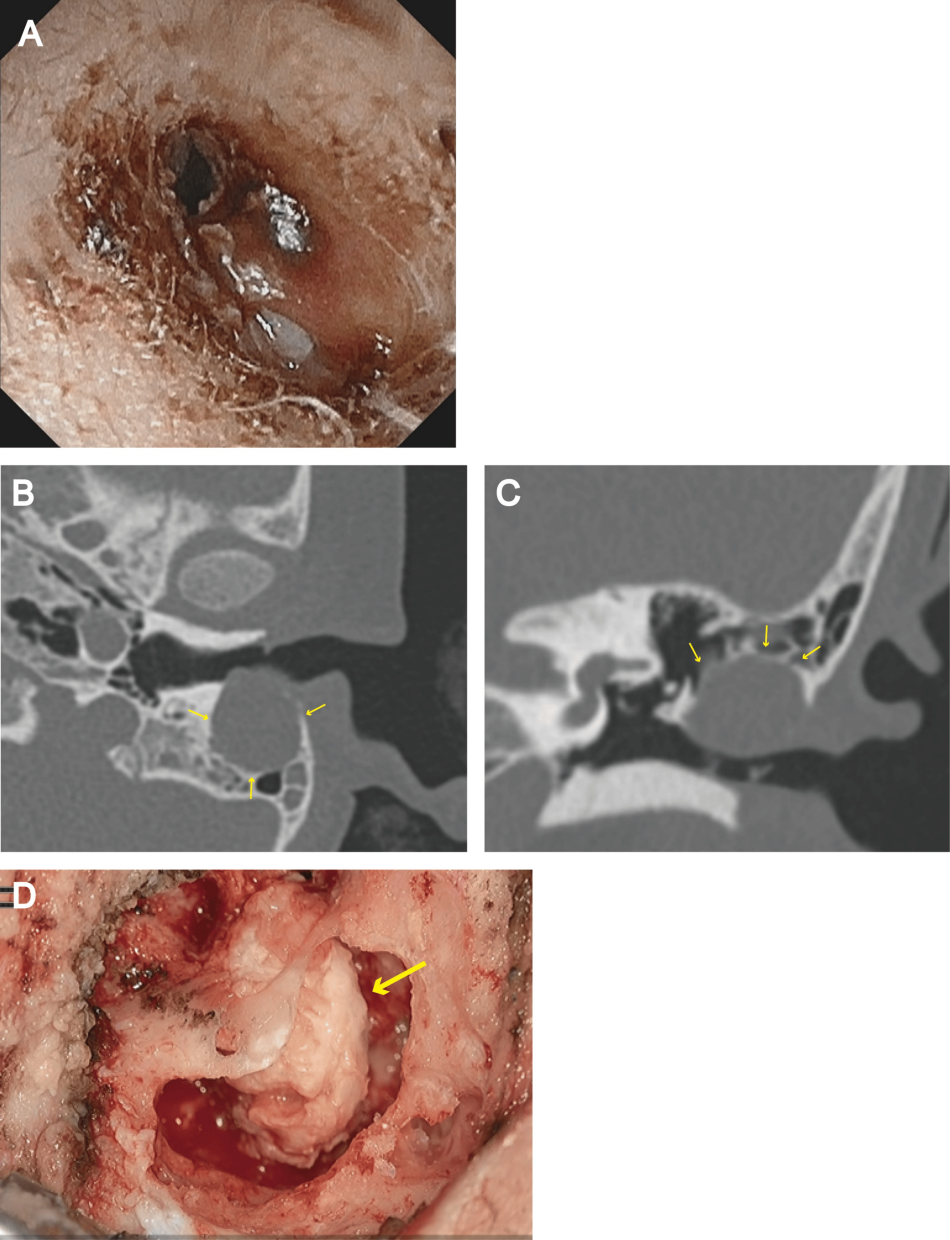
Preoperative and intraoperative findings. (A) Preoperative otoscopic findings. Tightly compacted granulation tissue almost filled the external auditory canal. Preoperative axial (B) and coronal (C) computed tomography (CT) images of the temporal bone. A round mass with a smooth, well-defined margin (yellow arrows) compressed and destroyed the posterior wall of the external auditory canal. The mass did not involve the mastoid tegmen, sigmoid sinus, or tympanic membrane. (D) Intraoperative findings. After removing the mastoid cortex, a cholesteatoma (yellow arrow) was found in the mastoid cavity.

A five-year-old girl presented to our hospital with swelling in the posterior wall of the left external auditory canal. The swelling was initially noted two months prior during a clinical visit for the removal of earwax. At that time, a smooth-surfaced bulge on the posterior wall of the external auditory canal was observed. However, it subsequently worsened, and granulation tissue formed on the surface. The patient did not have a history of ear discharge, ear infection, ear surgery, or other otologic complaints. Furthermore, the mother reported that the patient had not undergone ear cleaning. Otoscopic examination revealed tightly compacted granulation tissue that almost filled the entire external auditory canal, which impeded visualization of the left tympanic membrane (Figure 1A). Computed tomography (CT) of the temporal bone revealed a characteristic round mass that compressed and destroyed the posterior wall of the external auditory canal (Figure 1B-1C). The mass did not involve the mastoid tegmen, sigmoid sinus, or tympanic membrane (Figure 1B-1C).

Based on the CT findings and the lack of habitual ear cleaning, the patient was diagnosed with congenital EACC and underwent surgery. Intraoperatively, a defect in the posterior wall of the external auditory canal was found immediately after lifting the tympanomeatal flap via a retroauricular approach. After the mastoid cortex was removed, a closed-type cholesteatoma was found in the mastoid cavity, which was continuous with the skin surrounding the defect in the external auditory canal (Figure 1D). After mastoidectomy, the cholesteatoma was dissected along the matrix and completely removed. Subsequently, the bone defect in the posterior wall of the external canal was reconstructed using auricular cartilage and temporal fascia. The diagnosis of cholesteatoma was confirmed via pathological examination. At one postoperative year, CT did not show recurrence of cholesteatoma.

## Discussion

Pediatric EACC is a rare condition, with an incidence of 1.6 per 1,000 new otological patients in a single pediatric otology clinic in South Korea [8]. However, there has been an increase in the number of reported cases of pediatric EACC, particularly in Asian countries (Table 1) [4-6,8-12]. This apparent regional predominance may be related to the ear cleaning habit in Asia, which can cause repeated microtrauma to the external auditory canal and promote the development of acquired EACC. Nonetheless, few studies have clearly distinguished between congenital and acquired EACC in pediatric cases. To date, there have only been two reports of a clear diagnosis of congenital EACC; however, they did not explain the rationale behind this diagnosis (Table 1) [4,5]. Generally, congenital EACC is not diagnosed at an early stage because of a lack of symptoms.

**Table 1:** Reported English-language studies on pediatric external auditory canal cholesteatoma (EACC).

Authors	Year	Country	Number of pediatric patients	Distinction between congenital/acquired
Quantin et al. [5]	2002	France	1	Yes (congenital EACC)
Cheng et al. [9]	2005	Taiwan	1	No
Yoon et al. [8]	2008	Korea	9	No
Choi et al. [4]	2011	Korea	1	Yes (congenital EACC)
Kim et al. [11]	2014	Korea	8	No
Jang et al. [12]	2016	Korea	7	No
He et al. [10]	2019	China	35	No
Zhang et al. [6]	2024	China	44	No
Present case	2025	Japan	1	Yes (congenital EACC)

**Figure 2:**
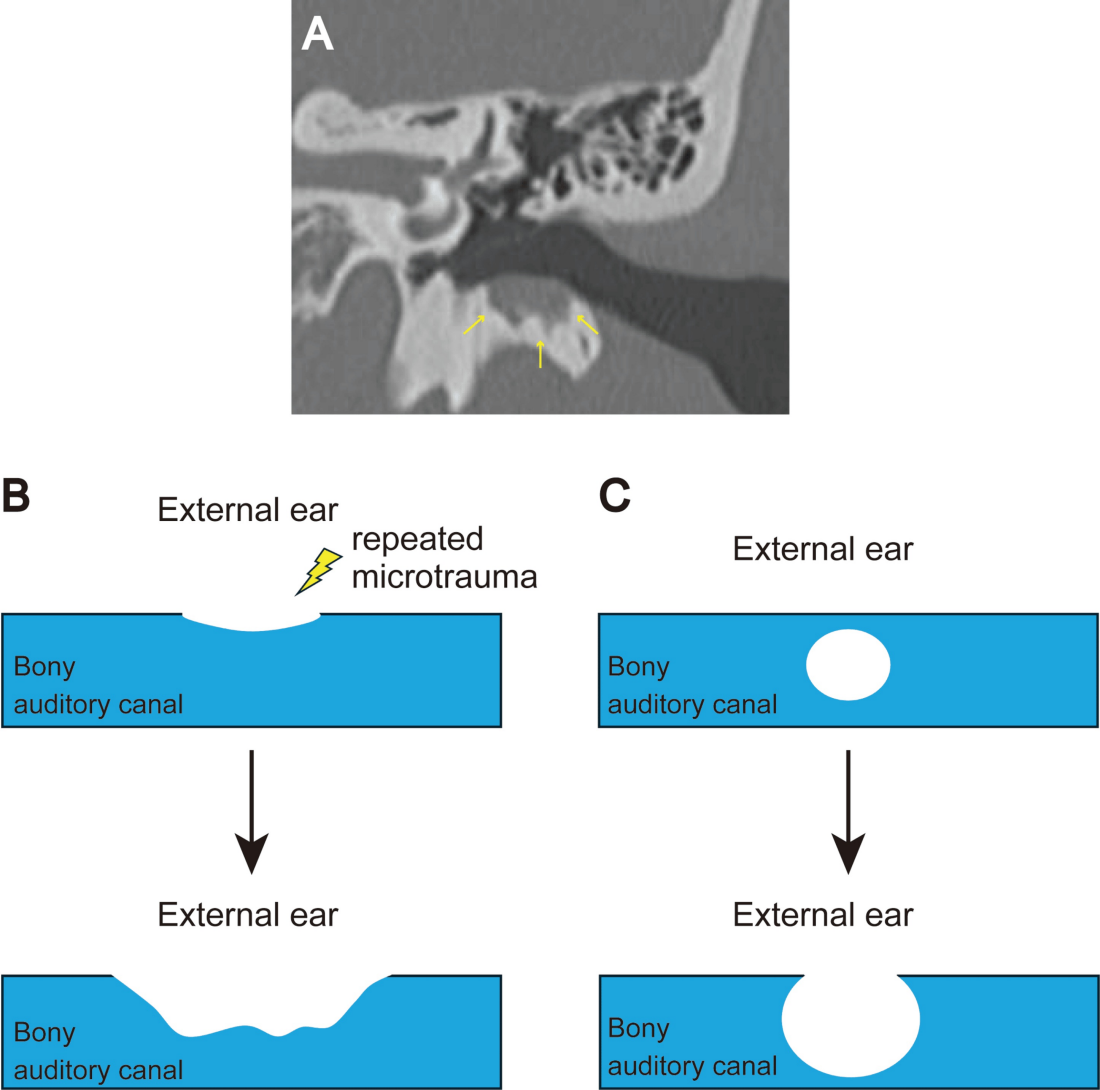
Comparison of CT findings in our case and a previous suspected case of acquired EACC, as well as our hypothesis regarding the between-form differences in CT findings. (A) Coronal CT images of a suspected case of acquired EACC in a 14-year-old boy. A saucer-shaped depression with an irregular margin was observed (yellow arrows). (B, C) Our hypothesis regarding the differences in CT findings between the depicted case and the current case. EACC: external auditory canal cholesteatoma.

In case the surface of a congenital EACC remains intact, it is relatively easy to differentiate it from acquired EACC based on clinical symptoms and otoscopic findings. Acquired EACC in pediatric cases typically presents as a focal skin disruption accompanied by underlying bony erosion and accumulation of keratin debris on otoscopy, with otorrhea and/or otalgia being the most common symptoms [6,8,12]. Contrastingly, congenital EACC in pediatric cases typically presents as stenosis of the external auditory canal with intact skin on otoscopy; furthermore, it is usually asymptomatic in the absence of a secondary infection. However, it is relatively difficult to distinguish between these forms once there is rupture or formation of inflammatory granulation tissue on the surface of a congenital EACC. In such cases, CT is a valuable diagnostic tool. In the present case, CT examination revealed a round depression with smooth margins (Figure 1B). Contrastingly, CT examination in a suspected case of acquired EACC revealed a saucer-shaped depression with irregular margins (Figure 2A). This difference may be attributed to differences in the underlying etiology. We hypothesize that in acquired EACC, repeated microtrauma initially causes a small defect in the external auditory canal (Figure 2B). This defect progressively deepens and expands, which ultimately results in a large defect with irregular margins (Figure 2B). Contrastingly, in congenital EACC, a round epithelial rest initially occurs within the external auditory canal (Figure 2C). Subsequently, it gradually grows and compresses the external auditory canal, with eventual protrusion beyond the canal (Figure 2C). Based on the aforementioned criteria, some of the previously reported cases of pediatric EACC that were simply labeled as EACC were likely to have been congenital cholesteatoma. For example, the CT image of Patient 1 in the article by Jang et al. revealed a typical smooth and round defect in the external auditory canal, which is strongly suggestive of congenital cholesteatoma [12]. Naturally, our proposed criteria have limitations, as they are based on a single case report. Additional cases are needed to validate these criteria.

In the present case, the congenital EACC was located in the posterior wall of the external auditory canal. However, congenital EACC can arise in various regions of the canal. For example, the cases reported by Choi et al. and Jang et al. involved lesions originating from the inferior wall of the canal [4,12]. Additionally, it is important to consider differential diagnoses of congenital tumors (e.g., teratomas, dermoid cysts, lipomas, and chondromas) [4].

As observed in the present case, extension of a congenital cholesteatoma into both the external auditory canal and mastoid cavity impedes the determination of whether the lesion originates from the external auditory canal or the mastoid cavity. Based on previous reports [2,13,14], congenital cholesteatomas originating from the mastoid tend to fill the mastoid cavity before invading adjacent structures, including the external auditory canal, mastoid tegmen, and sigmoid sinus. Among these adjacent structures, the external auditory canal is usually the last one to be eroded [2,13,14]. Therefore, in case there is invasion of the external auditory canal prior to complete occupation of the mastoid cavity, a diagnosis of congenital EACC should be considered. Similarly, Nagato et al. described a case of a round mass that destroyed the posterior wall of the external auditory canal without complete occupation of the mastoid cavity [3]. However, the lesion was diagnosed as a congenital cholesteatoma originating in the mastoid cavity. Given the aforementioned characteristic features of congenital cholesteatomas arising in the mastoid, the validity of this previous diagnosis is questionable.

Acquired EACC with small, localized lesions can typically be conservatively managed through regular microscopic debridement combined with topical antibiotic therapy. Contrastingly, congenital EACC usually requires surgical intervention since it is often not detected until the lesion protrudes beyond the external auditory canal.

## Conclusions

Pediatric congenital EACC is a rare clinical entity, which may be partly attributed to underreporting and diagnostic challenges. Congenital and acquired cholesteatomas have clinically important differences and therefore require careful differentiation. Congenital EACC should be considered in patients with external auditory canal stenosis, particularly in the absence of a history of trauma or infection. Furthermore, the diagnosis should be based on a comprehensive evaluation, including clinical examination, radiological imaging, and histopathological analysis. Prompt surgical resection is recommended to prevent lesion progression and erosion of adjacent anatomical structures. However, the proposed diagnostic considerations in this report are based on a single case, which is a clear limitation. Larger case series are needed to validate these findings and establish reliable diagnostic criteria for congenital EACC.
